# Species diversity, host preference and arbovirus detection of *Culicoides* (Diptera: Ceratopogonidae) in south-eastern Serbia

**DOI:** 10.1186/s13071-019-3292-3

**Published:** 2019-01-25

**Authors:** Ana Vasić, Nemanja Zdravković, Dragoș Aniță, Jovan Bojkovski, Mihai Marinov, Alexander Mathis, Marius Niculaua, Elena Luanda Oșlobanu, Ivan Pavlović, Dušan Petrić, Valentin Pflüger, Dubravka Pudar, Gheorghe Savuţa, Predrag Simeunović, Eva Veronesi, Cornelia Silaghi, the SCOPES AMSAR training group, Adriana Aniță, Ioana Alexandra Anton, Andrei Cimpan, Lavinia Ciucă, Luciana Crivei, Aleksandar Cojkić, Darko Davitkov, Vladimir Drašković, Bojan Gajić, Uroš Glavinić, Maria-Larisa Ivănescu, Mihaela Kavran, Andrei-Cristian Lupu, Raluca Mîndru, Daniela Porea, Radiša Prodanović, Oliver Radanović, Cristian Răileanu, Stefan Raileanu, Marko Ristanić, Constantin Roman, Ljubodrag Stanišić, Slavica Vaselek, Miloje Đurić

**Affiliations:** 1 Faculty of Veterinary Medicine, University of Belgrade, Belgrade, Serbia; 2 Institute of Infectology, Friedrich-Loeffler-Institute, Insel Riems, Germany; 3 Scientific Veterinary Institute of Serbia, Belgrade, Serbia; 4 Faculty of Veterinary Medicine of Iaşi, Iaşi, Romania; 5 Danube Delta National Institute for Research and Development, Tulcea, Romania; 6 National Centre for Vector Entomology, Institute of Parasitology, Vetsuisse Faculty, University of Zürich, Zürich, Switzerland; 7 Research Centre for Oenology Iaşi, Iaşi, Romania; 8 Faculty for Agriculture, University of Novi Sad, Novi Sad, Serbia; 9 Mabritec AG, Riehen, Switzerland; 10 Ernst-Moritz-Arndt-Universität, Greifswald, Germany

**Keywords:** *Culicoides* spp., BTV, SBV, Host preference, Serbia, Capacity building, Train the trainers concept

## Abstract

**Background:**

*Culicoides* (Diptera: Ceratopogonidae) is a genus of small biting midges (also known as “no-see ums”) that currently includes 1368 described species. They are proven or suspected vectors for important pathogens affecting animals such as bluetongue virus (BTV) and Schmallenberg virus (SBV). Currently little information is available on the species of *Culicoides* present in Serbia. Thus, the aim of this study was to examine species diversity, host preference and the presence of BTV and SBV RNA in *Culicoides* from the Stara Planina Nature Park in south-eastern Serbia.

**Results:**

In total 19,887 individual *Culicoides* were collected during three nights of trapping at two farm sites and pooled into six groups (Obsoletus group, Pulicaris group, “Others” group and further each group according to the blood-feeding status to freshly engorged and non-engorged). Species identification was done on subsamples of 592 individual *Culicoides* specimens by morphological and molecular methods (MALDI-TOF mass spectrometry and PCR/sequencing). At least 22 *Culicoides* species were detected. Four animal species (cow, sheep, goat and common blackbird) as well as humans were identified as hosts of *Culicoides* biting midges. The screening of 8291 *Culicoides* specimens in 99 pools for the presence of BTV and SBV RNA by reverse-transcription quantitative PCR were negative.

**Conclusions:**

The biodiversity of *Culicoides* species in the natural reserve Stara Planina was high with at least 22 species present. The presence of *C. imicola* Kieffer was not recorded in this area. *Culicoides* showed opportunistic feeding behaviour as determined by host preference. The absence of SBV and BTV viral RNA correlates with the absence of clinical disease in the field during the time of sampling. These data are the direct outcome of a training programme within the Institutional Partnership Project “AMSAR: Arbovirus monitoring, research and surveillance-capacity building on mosquitoes and biting midges” funded by the programme SCOPES of the Swiss National Science Foundation.

## Background

*Culicoides* (Diptera: Ceratopogonidae) is a genus of small biting midges (also known as “no-see ums”) that currently includes 1368 described species [1] in 32 subgenera [2]. They are important vectors of arboviruses of veterinary (bluetongue virus (BTV) [3, 4], Schmallenberg virus (SBV) [5], African horse sickness virus (AHSV) [3], epizootic haemorrhagic disease virus (EHDV) [6]) and medical importance (Oropouche virus) [7, 8]. *Culicoides* tend to blood-feed on and breed near domestic livestock and humans [9]. *Culicoides*-borne virus transmission in Europe is especially important for BTV which causes significant economic losses [10]. Even though *Culicoides imicola* Kieffer, one of the major BTV and AHSV vectors in Africa, southern Europe and Southeast Asia, seems to increase its distribution northwards [11]*,* the expansion of BTV into Europe has enforced a re-evaluation of the importance of Palaearctic *Culicoides* species as competent vectors [12]. The role of other than *C. imicola* species was proven for the first time in Europe in studies from Italy [13, 14]. The predominant Obsoletus complex and Pulicaris complex were implicated in BTV transmission during the outbreak of BTV in northern Europe in 2006 [15]. It was postulated that infected *Culicoides* individuals were introduced by transport within ship-containers or by transport of live animals from endemic regions in Africa [15, 16]. It is also described that *Culicoides* introduction can occur *via* meteorological conditions (such as wind) [17]. In 2011, SBV has been reported for the first time in Europe in cattle from Germany and the Netherlands, causing disease with fever, decreased milk production, diarrhea and malformed newborn animals [5]. It rapidly spread through Europe in 2012 and 2013 [18], and re-emerged in Germany in 2014 with high sequence identity of the isolated virus genome to the first SBV sample implicating possible persistence of virus within the insect vectors [19].

The investigation of species occurrence, diversity, and abundance of the genus *Culicoides* in south-eastern Europe and the Balkan Peninsula started after the first introduction of BTV into Bulgaria in 1999 revealing the presence of Obsoletus complex specimens (75%) followed by Pulicaris complex (16%) [20]. Subsequent outbreaks of BTV occurred and entomological studies were done in Albania [21], Bosnia and Herzegovina [22], Croatia [23], the former Yugoslav Republic of Macedonia (FYROM), Montenegro and Serbia [21, 24]. *Culicoides imicola* was not captured or reported in any of the above-mentioned studies.

Since the outbreak of BTV serotype 9 in Serbia in 2002, the country was free of BTV until August 2014 when a new outbreak of BTV serotype 4 occurred [25]. The state monitoring programme in 2015 consisted of insect trapping, identification and detection of viral genome in *Culicoides* samples [25].

To contribute further to the knowledge on *Culicoides* in Serbia, the aims of our study were: (i) to identify *Culicoides* species present in the area of Stara Planina Nature Park (south-east Serbia); (ii) to identify host species for the local *Culicoides* population by DNA characterization; and (iii) to screen for BTV and SBV RNA in the collected *Culicoides* specimens.

## Methods

### AMSAR project concept

The Swiss National Science Foundation provided funding for the SCOPES (Scientific co-operation between eastern Europe and Switzerland) project No. 160429, “Arbovirus Monitoring, Surveillance and Research-capacity building on mosquitoes and biting midges (AMSAR)”. This project was a trilateral institutional partnership aiming at capacity building and spreading knowledge between partner institutions from Switzerland, Romania and Serbia during 2015–2017. The goal of the project was to provide training to young scientists in Romania and Serbia who would be able to continue working in the field of medical and veterinary entomology. The innovative “train the trainers” concept was used for the first time in this field and as a result, knowledge was widely shared and disseminated [Silaghi C. AMSAR: a capacity building project based on the “Train the trainers” concept. ESOVE 2016, 2 –7.10.2016, Lisbon, Portugal]. Thus, the authors of this paper were participants of the project involved in practical field and laboratory investigations.

### Study area and description of stables

Stara Planina, located is south-eastern Serbia, is a nature reserve of the Ia protection category, i.e. strictly protected areas set aside to protect biodiversity and also possibly geological/geomorphical features, where human visitation, use and impacts are strictly controlled and limited to ensure protection of the conservation values according to the International Union for Conservation of Nature (IUCN).

The Stara Planina Nature Park is remote mountainous terrain (highest elevation Midzor peak, altitude 2169 m) with a high biodiversity of 1200 plant species (including 115 endemic, 100 strictly protected by State and 50 on the list of endangered species in Europe) and several animal species (116 butterflies, 46 amphibians and reptiles, 26 fish, 203 birds and 30 mammals) [26]. The autochthonous cattle breed “Busa” (approx. *n* = 100) is kept indoors at location A, in the area of Gornji Krivodol at an altitude of 886 m above sea level (43°6'37"N, 22°57'14"E) while location B is a private farm with two separate animal breeding locations for up to 50 goats indoors and up to 50 sheep outdoors in the village Kamenica at an altitude of 811 m (43°28'28"N, 22°21'21").

### Insect collection

Insects were collected overnight in 70% ethanol using Onderstepoort Veterinary Institute (OVI) traps with UV light as source of attraction [27]. At each location, two traps were placed, one inside and the other outside of the stable. At location A, two overnight samplings (4th and 6th July 2016) were done, and at location B there was one night of sampling (5th July 2016). At the time of sampling, the morning temperatures at 6:00 h on 4th, 5th and 6th July 2016 were 14 °C, 16 °C and 16 °C and the maximum daily temperatures at 16:00 h were 26 °C, 29 °C and 32 °C, respectively. There was no atmospheric precipitation, the average relative humidity was 60% and wind velocity was up to 7.5 km/h [28].

### Insect identification and sorting

All collected insects were separated into *Culicoides* specimens and other insects, which were discarded. From each trap, approximately 100 *Culicoides* individuals were randomly taken for morphological identification to the species level under a stereomicroscope with 10× and 20× magnification using the Interactive IIKC key [29, 30]. From the morphologically identified *Culicoides* specimens, the ones belonging to a species with an existing MALDI-TOF MS (Matrix Assisted Laser Desorption Ionization-Time of Flight Mass Spectrometry) spectrum were species confirmed by MALDI-TOF MS. Most of the remaining morphologically identified *Culicoides* specimens were identified by PCR followed by sequencing in order to obtain final species identifications. Additionally, some of the *Culicoides* specimens were tested with both MALDI-TOF MS and sequencing.

All remaining *Culicoides* specimens from each night of trapping were separated into three groups (Obsoletus group, Pulicaris group and “Others” group) and further into freshly engorged and non-engorged forming a total of six groups. From each of the six groups from the six traps, up to 5 pools with up to 100 individuals each (depending on availability) were formed. The final total was 99 pools with altogether 8291 individual *Culicoides* (see below).

### Identification of *Culicoides* species using molecular methods

DNA was extracted from the abdomens of the *Culicoides* with the GeneJET whole blood genomic DNA purification mini kit (Thermo Fisher Scientific, Waltham, USA) according to the manufacturer’s instructions for blood with the modification that insects were disrupted in phosphate-buffered saline (PBS) in a Tissue Lyser II (Qiagen, Hombrechtikon, Switzerland) with a 5 mm stainless steel bead at 30 Hz for one minute twice before 20 μl proteinase K was added to a total volume of 200 μl. The incubation at 56 °C was done overnight.

The quality and quantity of the obtained DNA was measured with Nanodrop ND-1000 spectrophotometer (Thermo Fisher Scientific, Waltham, USA).

PCR was performed targeting a 585 bp region of the mitochondrial *cox*1 gene to identify the insect species. The multiplex PCR kit (Qiagen, Hombrechtikon, Switzerland) was used with the following primers: 0.5 μl of 100 μM C1-J-1718 mod (5'-GGA GGA TTT GGA AAT TGA TTTG-3') and 0.5 μl of 100 μM C1-N-2191 mod (5'-GTA AAA TTA AAA TAT AAA CTT CTGG-3') in final reaction volumes of 50 μl. A plasmid containing the target sequence of *C. imicola* was used as positive control and sterile water as a negative control [31].

The Qiagen MinElute kit (Qiagen, Hombrechtikon, Switzerland) was used to purify amplicons, and sequencing was done at Synergene (Schlieren, Switzerland). Chromatograms were quality checked and edited with Finch TV (finchtv.software.informer.com*)* and compared against the GenBank database using BLASTn [32] and BOLD [33]. Similarities higher than 97% were considered as a species match.

For MALDI-TOF mass spectrometry, head and thorax of insects were prepared as previously described [34]. Identification of specimens was done on a Mass Spectrometry Axima*™* Confidence machine (Shimadzu-Biotech Corp., Kyoto, Japan) and the spectra were compared to the existing database [34].

### Blood-meal identification

A two-step approach was used for host identification in blood-fed individuals. First, all samples were screened with a multiplex PCR approach based on *cytb* polymorphisms using 0.5 μl of 100 μM of primers UNIV2 (5'-TGA GGA CAA ATA TCA TTY TGA GGR GC-3'), CAPRA (5'-TTA GAA CAA GAA TTA GTA GCA TGG CG-3'), OVIS (5'-GGC GTG AAT AGT ACT AGT AGC ATG AGG ATG A-3') and BOVIS (5'-TTA GAT GTC CTT AAT GGT ATA GTA G-3') [35] in final volumes of 50 μl to detect blood meals on cow, goat and sheep. In the case of negative samples, these were tested with a generic PCR targeting the *cytb* gene (primers Cytbf 5'-GAG GMC AAA TAT CAT TCT GAG G-3' and Cytbr 5'-TAG GGC VAG GAC TCC TCC TAG T-3') followed by sequencing with the sequencing primer 5'-GGA CTC CTC CTA GTT TGT T5G G-3' as previously described [36]. Products were purified, sequenced and analysed as described above.

### Pathogen detection by molecular methods

In order to determine RNA presence of SBV and BTV, RNA extraction was done from 99 pools of up to 100 *Culicoides* specimens the Gene JET RNA Purification kit (Thermo Fisher Scientific, Waltham, USA) following the manufacturer’s instructions. Insect tissue was disrupted in 300 μl lysis buffer with 3 mm bead in the Tissue Lyser II for 20–40 s at 30 Hz. Quality and quantity of RNA was measured with the Nanodrop ND-1000 spectrophotometer (Thermo Fisher Scientific, Waltham, USA). No sample was excluded because of low RNA quantity or quality (quantity ranged from 1.03 ng/μl to 665.75 ng/μl; quality (A260/280 ratio) from 1.62 to 2.45).

For BTV RNA detection, the iTaq universal probes one-step kit (Bio-Rad, Hercules, USA) was used with primers BTV_IVI_F (5'-TGG AYA AAG CRA TGT CAA A-3'), BTV_IVI_R (5'-ACR TCA TCA CGA AAC GCT TC-3'), and the probe BTV_IVI_P (FAM-5'-ARG CTG CAT TCG CAT CGT ACG C-3'-BHQ1) as previously described [37].

For SVB RNA detection, the primers used were SBV-S-382F (5'-TCA GAT TGT CAT GCC CCT TGC-3'), SBV-S-469R (5'-TTC GGC CCC AGG TGC AAA TC-3') and the fluorogenic probe was FAM-5'-TTA AGG GAT GCA CCT GGG CCG ATG GT-3'-BHQ1 [5].

## Results

### Insect trapping

A total of 19,887 individual *Culicoides* specimens were collected during three nights of trapping in a total of six traps at two different locations (A and B). The total number of collected individuals per trap/night, their morphological grouping and blood-feeding status are shown in Table 1. Altogether, 6396 specimens (32.2%) were from the Obsoletus group, 1833 (9.2%) from the Pulicaris group, 11,649 (58.6%) from the “Others” group, and 9 damaged specimens (0.04%) could not be attributed to any group because group specific features were missing.

**Table 1:** Total number of collected *Culicoides* individuals by group and blood feeding status at two farm sites (A and B) in Stara Planina, Serbia

Location	Day of collection	Trap placement	Total no. of *Culicoides* collected			Obsoletus group		Pulicaris group		“Others” group		*Culicoides* spp.^a^	
			Total *n* (%)	Engorged *n* (%)	Non-engorged *n* (%)	Engorged *n* (%)	Non-engorged *n* (%)	Engorged *n* (%)	Non-engorged *n* (%)	Engorged *n* (%)	Non-engorged *n* (%)	Engorged *n* (%)	Non-engorged *n* (%)
A: cattle	04.07.2016	Inside stable	187 (100)	18 (9.63)	169 (90.37)	1 (0.53)	97 (51.87)	1(0.53)	20 (10.70)	16 (8.56)	52 (27.81)	0 (0)	0 (0)
A: cattle	04.07.2016	Outside stable	3394 (100)	869 (25.60)	2525 (74.4)	131 (3.86)	1124 (33.12)	43 (1.27)	263 (7.75)	693 (20.42)	1136 (33.46)	2 (0.06)	2 (0.06)
A: cattle	06.07.2016	Inside stable	774 (100)	101 (13.05)	673 (86.95)	6 (0.78)	69 (8.91)	6 (0.78)	45 (5.81)	89 (11.50)	559 (72.22)	0 (0)	0 (0)
A: cattle	06.07.2016	Outside stable	10,435 (100)	3705 (35.5)	6730 (64.5)	411 (3.94)	1375 (13.18)	113 (1.08)	869 (8.33)	3181 (30.48)	4483 (42.96)	0 (0)	3 (0.03)
Subtotal A			14,790 (100)	4693 (31.73)	10,097 (68.27)	549 (3.71)	2665 (18.02)	163 (1.10)	1197 (8.09)	3979 (26.90)	6230 (42.12)	2 (0.02)	5 (0.04)
B: sheep and goats	05.07.2016	Inside stable	2694 (100)	1169 (43.39)	1525 (56.61)	648 (24.05)	861 (31.96)	99 (3.67)	137 (5.09)	421 (15.63)	526 (19.52)	1 (0.04)	1 (0.04)
B: sheep and goats	05.07.2016	Outside stable	2403 (100)	59 (2.45)	2344 (97.55)	26 (1.08)	1647 (68.54)	8 (0.33)	229 (9.53)	25 (1.04)	468 (19.48)	0 (0)	0 (0)
Subtotal B			5097 (100)	1228 (24.09)	3869 (75.91)	674 (13.22)	2508 (49.21)	107 (2.10)	366 (7.18)	446 (8.75)	994 (19.5)	1 (0.02)	1 (0.02)
Total			19,887 (100)	5921 (29.77)	13,966 (70.23)	1223 (6.15)	5173 (26.01)	270 (1.36)	1563 (7.86)	4425 (22.25)	7224 (36.33)	3 (0.01)	6 (0.03)

^a^*Culicoides* spp.: grouping was not possible because group specific features were missing due to samples damage

As shown in Table 1, 14,790 (74.4%) individuals were collected at location A (cattle farm). Higher numbers of *Culicoides* specimens (*n* = 13,829; 93.5%) were trapped outside the stables rather than inside (*n* = 961; 6.5%).

At location B (goat/sheep farm), 5097 (25.6%) individual *Culicoides* were collected. Interestingly, the number of *Culicoides* specimens trapped inside the stable (*n* = 2694; 52.85%) was similar to that sampled outside of the stable (*n* = 2403; 47.14%).

Overall 5921 (29.8%) individual *Culicoides* were freshly engorged (4693 at location A and 1228 at location B) and 13,966 (70.2%) were non-engorged (10,097 at location A and 3869 at location B).

### Individual insect identification

Insect identification to the species level by morphology, MALDI-TOF MS and sequencing was completed on 592 *Culicoides* specimens, 393 from location A and 199 from location B (Table 2).

**Table 2:** Number of identified *Culicoides* to species level by MALDI-TOF mass spectrometry and PCR/sequencing per location in Stara Planina, Serbia. Some individuals identified by more than one method

Species	No. of identified individuals	Location A	Location B	*Culicoides* group	No. of identifications by MALDI-TOF	No. of identifications by sequencing
*C. achrayi*	2	2	0	“Others”	0	2
*C. circumscriptus*	3	3	0	“Others”	1	2
*C. clastrieri*	1	1	0	“Others”	0	1
*C. deltus*	5	2	3	“Others”	3	2
*C. dewulfi*	1	1	0	Obsoletus	0	1
*C. fagineus*	1	0	1	“Others”	0	1
*C. fascipennis*	71	49	22	“Others”	16	64
*C. festivipennis*	11	11	0	“Others”	7	4
*C. furcillatus*	11	3	8	“Others”	1	10
*C. kibunensis*	1	1	0	“Others”	0	1
*C. lupicaris*	31	21	10	Pulicaris	22	14
*C. newsteadi*	7	7	0	Pulicaris	1	6
*C. obsoletus/scoticus*	7	2	5	Obsoletus	0	0
*C. obsoletus*	69	39	30	Obsoletus	61	17
*C. pallidicornis*	4	4	0	“Others”	2	4
*C. parotti*	2	2	0	“Others”	0	2
*C. picturatus*	19	18	1	“Others”	0	19
*C. pulicaris*	3	2	1	Pulicaris	3	1
*C. punctatus*	11	11	0	Pulicaris	5	6
*C. salinarius*	2	2	0	“Others”	0	2
*C. scoticus*	150	61	89	Obsoletus	142	15
*C. simulator*	15	12	3	“Others”	0	15
*C. subfasciipennis*	10	10	0	“Others”	0	10
*Culicoides* spp.^a^	155	129	26		0	10
Total	592	393	199		264	209

^a^Identification to *Culicoides* spp. done as a combination of morphological identification result, indefinite sequencing results (poor identity or ambivalent result) and MALDI- TOF mass spectrometry results. Specimens defined as *C. obsoletus/scoticus* were identified by morphological identification, while *C.obsoletus* and *C. scoticus* were confirmed by molecular methods which enables species identification

All 592 *Culicoides* specimens were morphologically identified and all specimens without a confirmed identification by molecular methods (PCR/sequencing and/or MALDI-TOF MS) were classified as *Culicoides* spp. (155 in total). Using MALDI-TOF MS technique we identified up to 264 specimens at the species level, whereas 209 specimens were identified by sequencing. Some specimens were identified with more than one method.

Altogether 22 *Culicoides* species were recorded in the examined locations. As shown in Table 2, *C. deltus* Edwards, *C. fasciipennis* Staeger, *C. furcillatus* Callot, Kremer & Paradis, *C. lupicaris* Downes & Kettle, *C. obsoletus*, *C. picturatus* Kremer & Deduit, *C. pulicaris* Linnaeus, *C. scoticus* and *C. simulator* Edwards, were present on both the cow farm (location A) and the sheep/goat farm (location B). *Culicoides achrayi* Kettle & Lawson, *C. circumscriptus* Kieffer, *C. clastieri* Callot, Kremer & Deduit, *C. dewulfi* Goetghebuer, *C. festivipennis* Kieffer, *C. kibunensis* Tokunaga, *C. newsteadi* Austen, *C. pallidicornis* Kieffer, *C. parotti* Kieffer, *C. punctatus* Meigen, *C. salinarius* Kieffer and *C. subfasciipennis* Kieffer were present only at location A, while *C. fagineus* Edwards was present only at location B.

### Blood-meal identification

Out of 152 sequences of *Culicoides* samples for host preference analysis, 103 originated from location A while 49 were from location B. The blood hosts were identified in 14 *Culicoides* species and in 43 specimens identified as *Culicoides* spp. Alltogether 5 blood host species were confirmed. Overall, cows (*Bos taurus*) (*n* = 96), goats (*Capra hircus*) (*n* = 47), humans (*Homo sapiens*) (*n* = 6), sheep (*Ovis aries*) (*n* = 2) and common blackbirds (*Turdus merula*) (n=1) were identified as hosts (Table 3). Cows (*n* = 96) and blackbird (*n* = 1) were identified only at location A; sheep (*n* = 2) were identified only in location B, while goats were identified as hosts at location A (*n* = 2) and location B (n = 45). Human hosts were also found at location A (*n* = 4) and location B (*n* = 2).

**Table 3:** Identified blood hosts per *Culicoides* species and location

*Culicoides* species	Cow	Goat	Sheep	Blackbird	Human
*C. achrayi*	1	–	–	–	–
*C. circumscriptus*	–	–	–	–	–
*C. fascipennis*	31	7	1	–	–
*C. festivipennis*	1	–	–	–	–
*C. furcillatus*	–	2	–	–	1
*C. lupicaris*	4	2	–	–	–
*C. newsteadi*	1	–	–	–	–
*C. obsoletus*	5	7	1	–	–
*C. obsoletus/scoticus*	0	2	–	–	1
*C. pallidicornis*	2	–	–	–	–
*C. picturatus*	7	–	–	–	1
*C. punctatus*	4	–	–	–	–
*C. salinarius*	1	–	–	–	–
*C. scoticus*	1	18	–	–	2
*C. subfasciipennis*	5	–	–	–	1
*Culicoides* spp.^a^	33	9	–	1	–
Total	96	47	2	1	6
Total per location A	96	2	–	1	4
Total per location B	0	45	2	–	2

^a^Individual insects which were morphologically confirmed to be *Culicoides*, but species identification could not be concluded by MALDI-TOF mass spectrometry and/or PCR/sequencing

### Pathogen detection

A total of 8291 *Culicoides* were screened for BTV and SBV RNA presence. The number of individual *Culicoides* per group and feeding status is shown in Table 4. Out of 99 pools, of six different groups in total (Obsoletus group, *n* = 36; Pulicaris group, *n* = 20; “Others” group, *n* = 43) of which engorged (Obsoletus group, 14 pools; Pulicari*s* group, 5 pools; “Others” group, 17 pools) and non-engorged (Obsoletus group, 22 pools; Pulicaris group, 15 pools; “Others” group, 26 pools). Among the tested samples there were no positive results for BTV and SBV viral RNA by RT-qPCR (Table 4).

**Table 4:** *Culicoides* used for detection of BTV and SBV RNA in pools

Location, date and trap position	Total *N* (*n*)	Obsoletus group		Pulicaris group		“Others” group		SBV RT-qPCR	BTV RT-qPCR
		Engorged *N* (*n*)	Non-engorged *N* (*n*)	Engorged *N* (*n*)	Non-engorged *N* (*n*)	Engorged *N* (*n*)	Non-engorged *N* (*n*)		
A: 04.07.2016 inside	5 (86)	1 (1)	1 (40)	0 (0)	1 (12)	1 (6)	1 (27)	Negative	Negative
A: 04.07.2016 outside	21 (1887)	2 (105)	5 (500)	1 (38)	3 (244)	5 (500)	5 (500)	Negative	Negative
A: 06.07.2016 inside	11 (665)	1 (5)	1 (54)	1 (3)	1 (39)	1 (56)	6 (508)	Negative	Negative
A: 06.07.2016 outside	25 (2403)	4 (306)	5 (500)	1 (97)	5 (500)	5 (500)	5 (500)	Negative	Negative
B: 05.07.2016 inside	22 (1134)	5 (500)	5 (500)	1 (96)	2 (132)	4 (399)	5 (489)	Negative	Negative
B: 05.07.2016 outside	15 (2116)	1 (13)	5 (500)	1 (4)	3 (219)	1 (7)	4 (391)	Negative	Negative
Total	99 (8291)	14 (930)	22 (2094)	5 (238)	15 (1146)	17 (1468)	26 (2415)	Negative	Negative

*Abbreviations*: *N* Total number of pools, *n* number of individual *Culicoides*

## Discussion

In the past decade the Balkan Peninsula has encountered several outbreaks of BTV [24], and SBV activity was reported in 2013 [38]. Following these events, several studies were conducted in Bulgaria and Croatia to determine the abundance and species composition of *Culicoides* vectors [20, 23]. Even though there is a *Culicoides* monitoring programme [25] in Serbia, the data on abundance and species composition are scarce. The results of the Serbian *Culicoides* monitoring programme in 2015/2016 revealed the presence of *Culicoides* spp. from spring (April) to late autumn (December) [39]. Results of the present study showed that on both locations at Stara Planina Nature Park, *Culicoides* were present in large numbers, which is in correlation with the results of *Culicoides* collections in the neighbouring Bulgaria [20, 40]. The variation in number of collected *Culicoides* between two sampling dates at location A might have influence on the likelihood of collection of SBV- and BTV-positive specimens. This variation in numbers might have occurred due to altered microclimatic conditions between two sampling nights. Among the collected *Culicoides*, the morphological group “Others” was the most abundant (*n* = 11,649), followed by the Obsoletus group (*n* = 6396) and Pulicaris group (*n* = 1833). The highest number of individuals belonging to the Obsoletus group was recorded in Bosnia and Herzegovina [22], Croatia [23] and Romania [41]; however, this was not the case in our study. Since our study was completed in geographically close locations, we cannot generalize the group composition of *Culicoides* to larger territories in Serbia. Furthermore, in a study from Switzerland the group composition changed with different altitudes, revealing a higher abundance of *Culicoides* species that belong to “Others” at high altitudes [42].

We identified 22 *Culicoides* species. To our knowledge, there are no published data from neighboring countries (Greece, Croatia, and Bosnia and Herzegovina) on the presence of *C. clastieri*, *C. deltus*, *C. lupicaris*, *C. picturatus*, *C. salinarius*, *C. simulator* and *C. subfascipennis*, and *C. clastrieri*, *C. lupicaris* and *C. picturatus* were not recorded in Bulgaria. High *Culicoides* species diversity was recorded in Bulgaria with differences observed in species composition between two studies. This is probably due to habitat characteristics or availability of preferred blood hosts [20, 40]. In Croatia [23], the presence of *C. circumscriptus*, *C. fasciipennis*, *C. fagineus*, *C. haranti* Rioux, Descous & Pech, *C. obsoletus*, *C. paolae* Boorman, *C. pulicaris*, *C. punctatus*, *C. scoticus* and *C. seavanicus* Kieffer was determined and these results partially correlate with our findings. In Greece, 39 *Culicoides* species were found, and the findings differed according to the geographical area of the country [43]. Among these species, only 15 were found in our study, and 24 species detected in a study from Greece were not present in the sampled locations of Stara Planina Nature Park. Interestingly, *C. impunctatus* Goetghebuer was found in Bulgaria and Greece, but not in our study, possibly due to the sampling period. Our results did not show the presence of *C. imicola*, the main BTV vector in the Mediterranean basin, which is in agreement with findings in Albania [21], Bosnia and Herzegovina [22] and Bulgaria [20]. This implies the role of species other than *C. imicola* in the transmission cycle of BTV in the investigated locations in Serbia. Another study in northern Europe also identified that not a single specimen of *C. imicola* was detected amongst 100,000 *Culicoides* collected in France, Belgium and Luxemburg [44].

To the best of our knowledge, we describe the first data of host analysis for *Culicoides* in Serbia. Our results suggest for most species identified in this study have a mammophilic feeding behaviour, but interestingly, blood of a bird was recorded in one of the samples. Other domestic animals such as dogs and cats were also present at the sampling locations. The choice of animal host depends on intrinsic host preference of the insect species and host availability [45]. Opportunistic feeding tendencies in mammophilic biting midges for animals nearby were previously reported [35], which is also the observation in our study (location A - cow, location B - sheep and goat, as well as human hosts at both locations).

None of the tested *Culicoides* pools was positive for RNA of BTV and SBV. This finding is in relation with the absence of clinical cases in 2016 in the area of Stara Planina Nature Park (www.oie.int).

All results, discussions and conclusions presented here are a direct outcome from the capacity building project AMSAR based on the multiplying effect by “training-the-trainers” concept which has thus proven to be a successful scheme of capacity building in vector entomology.

## Conclusions

The biodiversity of *Culicoides* species in Stara Planina Nature Park is high and at least 22 species are present. *Culicoides imicola* was not recorded in this area. *Culicoides* showed opportunistic feeding behaviour as determined by host preference. The absence of SBV and BTV viral RNA correlates with the absence of clinical disease in the field during the time of sampling.
